# Propofol Reduces Lipopolysaccharide-Induced, NADPH Oxidase (NOX_**2**_) Mediated TNF-****α**** and IL-6 Production in Macrophages

**DOI:** 10.1155/2013/325481

**Published:** 2013-11-26

**Authors:** Tao Meng, Jingya Yu, Zhen Lei, Jianbo Wu, Shuqin Wang, Qiyu Bo, Xinyu Zhang, Zhiyong Ma, Jingui Yu

**Affiliations:** ^1^Department of Anesthesiology, Qilu Hospital of Shandong University, Shandong University, No. 107 Wen Hua Xi Road, Jinan, Shandong 250012, China; ^2^Department of Pathogeny Biology, Shandong University School of Medicine, No. 44 Wen Hua Xi Road, Jinan, Shandong 250012, China; ^3^Department of Cardiology, Qilu Hospital of Shandong University, Shandong University, No. 107 Wen Hua Xi Road, Jinan, Shandong 250012, China

## Abstract

During an infection, lipopolysaccharide (LPS) stimulates the production of reactive oxygen species (ROS), which is mediated, in large part, by nicotinamide adenine dinucleotide phosphate (NADPH) oxidases (NOXs); NOX_2_ is the major NOX isoform found in the macrophage cell membrane. While the immunomodulatory activity of propofol is highly documented, its effect on the LPS-induced NOX_2_/ROS/NF-*κ*B signaling pathway in macrophages has not been addressed. In present study, we used murine macrophage cell line RAW264.7 pretreated with propofol and stimulated with LPS. IL-6 and TNF-*α* expression, ROS production, and NOX activity were determined. Results showed that propofol attenuated LPS-induced TNF-*α* and IL-6 expression. Moreover, LPS-stimulated phosphorylation of NF-*κ*B and generation of ROS were weakened in response to propofol. Propofol also reduced LPS-induced NOX activity and expression of gp91phox and p47phox. We conclude that propofol modulates LPS signaling in macrophages by reducing NOX-mediated production of TNF-*α* and IL-6.

## 1. Introduction

Macrophages respond to invading microbes at multiple levels, including regulation of immune mediator secretion, microbial killing, pyroptosis, and adaptive immune instruction [1, 2]. During an infection, LPS is released into circulation, promoting the production of reactive oxygen species (ROS) in macrophages [3]. Despite their well-established cytotoxic activities [4], recent studies have shown that ROS are immunomodulatory agents that can enhance the immune response to an infection [5, 6]. Nicotinamide adenine dinucleotide phosphate (NADPH) oxidases (NOXs) are major sources of ROS in immune cells [7]. NOX_2_, a highly regulated membrane-bound enzyme, is the predominant isoform in macrophages and the major source of LPS-induced ROS in these cells [8]. NOX_2_ is composed of the transmembrane heterodimers gp91^phox^ and p22^phox^ (known collectively as cytochrome b_558_), and four regulatory cytosolic subunits—p40^phox^, p47^phox^, p67^phox^, and the small GTPase, Rac2. p47^phox^, and cytochrome b_558_ comprise the minimal functional subunit of NOX_2_ [9].

Propofol (2,6-diisopropylphenol) is a potent sedative and hypnotic agent that exhibits anti-inflammatory and antioxidant activity. In particular, propofol reduces the release of inflammatory mediators such as IL-6 and TNF-*α* and inhibits ROS in phagocytic cells [10–12]. However, the effect of propofol treatment on NADPH oxidases and ROS production in macrophages has not been studied. Here, we investigate the effect of propofol pretreatment on LPS-induced changes to ROS levels, NF-*κ*B phosphorylation, and NADPH oxidase activity in murine macrophage cell line RAW 264.7.

## 2. Materials and Methods

### 2.1. Cell Culture

The mouse macrophage cell line RAW264.7 was obtained from American Type Culture Collection (Manassas, VA). The cells were cultured at 37°C under 5% CO_2_ in DMEM supplemented with 10% FCS (Invitrogen-Life Technologies), 100 U/mL penicillin, and 100 *μ*g/mL streptomycin. Propofol (Sigma, MO, USA) was freshly dissolved in dimethyl sulfoxide (DMSO) (Sigma, MO, USA) for each experiment in an opaque tube to protect it from light. Control macrophages were treated with DMSO only. LPS (Sigma, MO, USA) was used at a final concentration of 100 ng/mL.

### 2.2. Detection of Cytokine Production

A total of 2 × 10^5^ macrophages were seeded into 24-well plates and incubated overnight. Cells were treated with DMSO or propofol for 40 min and then stimulated with LPS for 8 h. TNF-*α* and IL-6 concentration in culture was measured by an ELISA kit (Endogen, Woburn, MA) following the manufacturer's instructions. All operations were performed at room temperature. Absorbance at 450 nm for standards and samples, performed in duplicate, was measured using a Varioskan Flash multifunction plate reader (Thermo Scientific).

### 2.3. Analysis of Cytokine mRNA Levels Using RT-PCR

RAW264.7 macrophages were pretreated with DMSO or propofol for 40 min and then stimulated with LPS for 2 h. Total RNA was extracted with TRIzol reagent, according to the manufacturer's instructions (Invitrogen). A Light Cycler (ABI PRISM 7000) and a SYBR RT-PCR kit (Takara) were used for quantitative real-time PCR analysis. Specific primers used were 5′-GCCACCACGC-TCTTCTGTCT-3′ (sense) and 5′-TGAGGGTCTGGGCCA-TAGAAC-3′ (antisense) for TNF-*α*; 5′-ACAACCACGGCCTTCCCTAC-3′ (sense) and 5′-CATTTCCACGAT-TTCCCAGA-3′ (antisense) for IL-6; and 5′-TGTTACCAACTGGGACGACA-3′ (sense) and 5′-CTGGGTCATCTTTTCACGG T-3′ (antisense) for *β*-actin. Data are normalized to *β*-actin expression in each sample. Amplification, detection, and data analysis involved the use of the iCycler Real-Time PCR system (Bio-Rad Laboratories).

### 2.4. Detection of Akt and Nuclear NF-*κ*B Activation

RAW264.7 macrophages were pretreated with DMSO or propofol for 40 min and then stimulated with LPS for 60 min. Cells (5 × 10^6^) were harvested, resuspended in 50 *μ*L of lysis buffer A (10 mM Hepes, 10 mM KCl, 0.1 mM MgCl_2_, 0.1 mM EDTA, 2 *μ*g/mL leupeptin, 2 *μ*g/mL pepstatin, and 0.5 mM PMSF, pH 7.9), incubated on ice for 10 min and centrifuged for 10 min, at 800 ×g at 4°C. The supernatant was considered the cytoplasmic fraction. The pellet (nuclei) was washed with buffer A and nuclear proteins were extracted in presence of 50 *μ*L of buffer B (10 mM Hepes, 400 mM NaCl, 1.5 mM MgCl_2_, 0.1 mM EDTA, 2 *μ*g/mL leupeptin, 2 *μ*g/mL pepstatin, and 0.5 mM PMSF, pH 7.9). Nuclear and cytoplasmatic extracts were then analyzed for protein content and stored at −80°C. Equal amounts of protein were resolved using 10% SDS-PAGE and electrotransferred onto nitrocellulose membrane (Amersham Biosciences, NJ, USA). The membrane was blocked with TBS with 5% nonfat milk for 2 h at room temperature, washed in TBS-T 3 times for 10 min, and incubated with primary antibody at 4°C overnight. The primary antibodies were as follows: NF-*κ*B (1 : 1000, Cell Signaling Biotechnology), phosphorylated NF-*κ*B (Ser536) (1 : 500, Cell Signaling Biotechnology), Akt (1 : 1000, Santa Cruz Biotechnology), phosphorylated Akt (Ser 473) (1 : 500, Santa Cruz Biotechnology), and *β*-actin (1 : 2000, Cell Signaling Biotechnology). The membrane was then incubated with a secondary antibody. Protein bands were detected with an enhanced chemiluminescence kit and captured by Image-Pro Plus 6.0.

### 2.5. Measurement of Superoxide Production

The oxidative fluorescent dye hydroethidine (DHE) (Beyotime, Beijing) was used to evaluate levels of superoxide as described previously [13, 14]. Hydroethidine is cell permeable and in the presence of superoxide becomes oxidized to form ethidium bromide, which is a fluorescent DNA intercalator. This method provides sensitive detection of superoxide levels in cells. After a 40 min incubation with propofol or DMSO, cells were stimulated with LPS for 40 min then stained with 2 *μ*M DHE for 30 min at 37°C in the dark. Nuclei were stained with DAPI. Finally, cell samples were analyzed using an Olympus IX71 fluorescence microscope (Olympus, Tokyo, Japan).

### 2.6. NADPH Oxidase Activity Assay

NADPH oxidase activity was measured using an assay kit (GENMED, Beijing). After a 40 min treatment with propofol or DMSO, cells were stimulated with LPS for 6 h and then harvested. The supernatants were transferred to a tube, to which specific substrate conjugates for oxidase were added. NADPH oxidase activity was determined by spectrophotometry (Thermo Scientific) at 340 nm.

### 2.7. Determination of NADPH Oxidase Expression

Macrophages were pretreated with DMSO or propofol for 40 min and then stimulated with LPS for 8 h. The primary antibodies were as follows: NADPH oxidase p22^phox^ (1 : 1000, Santa Cruz Biotechnology, Santa Cruz, CA), NADPH oxidase gp91^phox^ (1 : 1000, Santa Cruz Biotechnology, Santa Cruz, CA), NADPH oxidase p47^phox^ (1 : 500, Santa Cruz Biotechnology, Santa Cruz, CA), and *β*-actin (1 : 2000, Cell Signaling Biotechnology).

### 2.8. Statistical Analysis

All data are presented as means ± SD. SPSS for Windows v.16.0 (SPSS Inc., Chicago, IL, USA) was used for statistical analysis. Analysis was performed using two-way analysis of variance. The *P* values < 0.05 were considered statistically significant.

## 3. Results

### 3.1. Effect of Propofol on LPS-Induced TNF-*α* and IL-6 Expression

**Figure 1:**
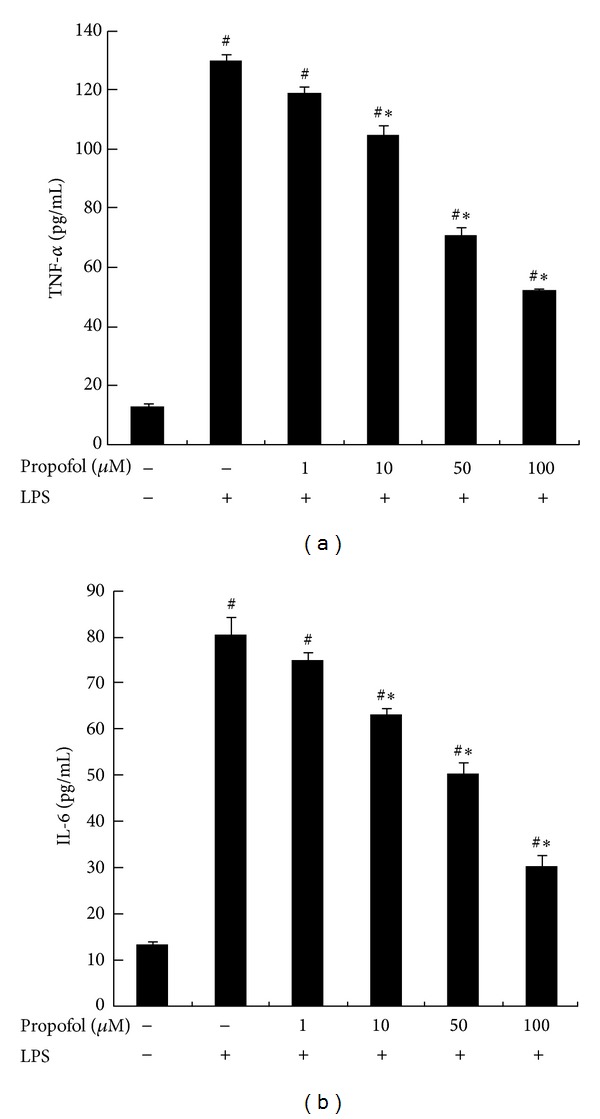
Effect of propofol on LPS-induced TNF-*α* and IL-6 secretion. Macrophages were pretreated with dimethyl sulfoxide (DMSO) or 1 *μ*M, 10 *μ*M, 50 *μ*M, and 100 *μ*M propofol for 40 min and stimulated with 100 ng/mL LPS for 8 h. The concentrations of TNF-*α* and IL-6 in culture supernatants were measured by ELISA. Each value represents the means ± SD for *n* = 4. # and ∗ indicate statistically significant differences (*P* < 0.05) between propofol and LPS treated and LPS only treated groups, respectively.

**Figure 2:**
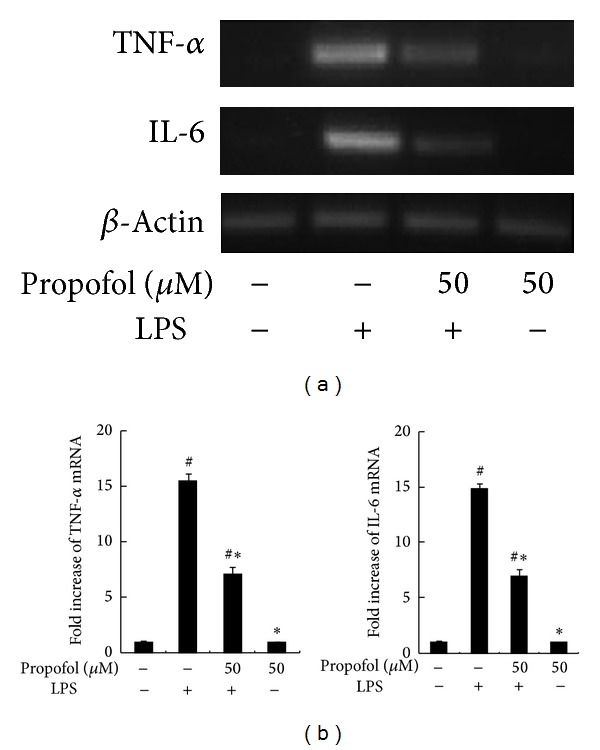
Effect of propofol on LPS-induced TNF-*α* and IL-6 expression. (a) RAW264.7 macrophages were pretreated with dimethyl sulfoxide (DMSO) or 50 *μ*M propofol for 40 min and then stimulated with LPS for 2 h. Steady state mRNA levels of TNF-*α* and IL-6 were examined by RT-PCR. (b) The levels of TNF-*α* and IL-6 mRNA were quantified by measuring band intensities and shown as fold increase relative to *β*-actin mRNA levels. Each value represents the means ± SD for *n* = 4. # and ∗ indicate statistically significant differences (*P* < 0.05) between propofol and LPS treated and LPS only treated groups, respectively.

Low levels of TNF-*α* and IL-6 mRNA were detected in untreated macrophages. LPS treatment (100 ng/mL) induced a significant increase in cellular TNF-*α* and IL-6 levels (*P* < 0.05). However, propofol pretreatment reduced LPS-induced TNF-*α* and IL-6 expression by 20.01 ± 5.4% (*P* < 0.05), 46.15 ± 6.8% (*P* < 0.05), and 61.53 ± 10.2% (*P* < 0.05) in response to 10 *μ*M, 50 *μ*M, and 100 *μ*M propofol, respectively (Figure 1). Treatment of macrophages with a therapeutic concentration of propofol (50 *μ*M) decreased LPS-induced production of TNF-*α* and IL-6 mRNA. However, 50 *μ*M propofol alone (i.e., without LPS treatment) did not affect TNF-*α* and IL-6 mRNA expression (Figures 2(a) and 2(b)).

### 3.2. Effect of Propofol on LPS-Induced Nuclear NF-*κ*B and Akt Phosphorylation

**Figure 3:**
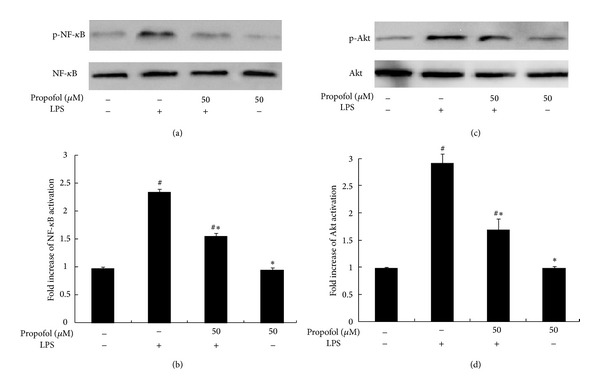
Effect of propofol pretreatment on LPS-induced phosphorylation of nuclear NF-*κ*B and Akt. (a), (c) The cells were pretreated with dimethyl sulfoxide (DMSO) or propofol for 40 min and then stimulated with LPS for 1 h. Nuclear NF-*κ*B and Akt activation, indicated by phosphorylation, was analyzed by Western blotting on whole cell lysates. (b), (d) The levels of phosphorylated NF-*κ*B (p-NF-*κ*B) and phosphorylated Akt (p-Akt) were quantified by measuring band intensities and represented as fold change over total NF-*κ*B and Akt. Each value represents the means ± SD for *n* = 4. # and ∗ indicate statistically significant differences (*P* < 0.05) between propofol and LPS treated and LPS only treated groups, respectively.

After treatment with 100 ng/mL LPS for 1 h, a significant increase in NF-*κ*B and Akt phosphorylation was observed. However, 50 *μ*M propofol pretreatment reduced the level of NF-*κ*B and Akt phosphorylation. Similar to its effect on TNF-*α* and IL-6, propofol treatment alone did not alter NF-*κ*B and Akt phosphorylation (Figures 3(a)–3(d)).

### 3.3. Effect of NF-*κ*B Inhibitor on LPS-Induced TNF-*α* and IL-6 Expression

**Figure 4:**
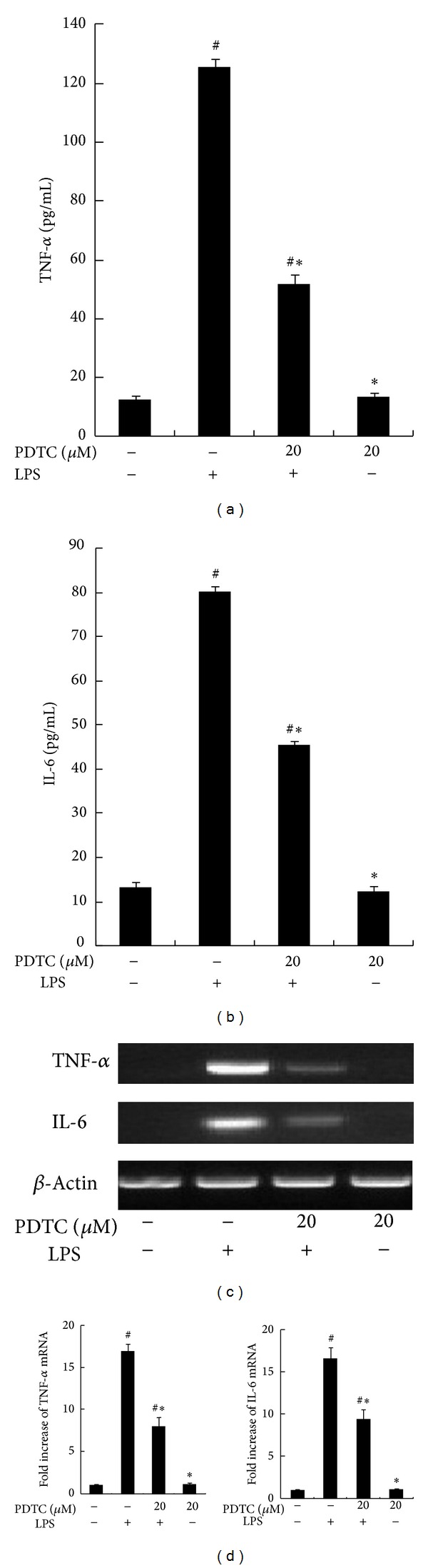
Effect of NF-*κ*B Inhibitor on LPS-induced TNF-*α* and IL-6 expression. RAW264.7 macrophages were pretreated with dimethyl sulfoxide (DMSO) or 20 *μ*M pyrrolidine dithiocarbamate (PDTC) for 1 h and stimulated with 100 ng/mL LPS for 8 h. The concentrations of TNF-*α* and IL-6 in culture supernatants were measured by ELISA. (c) RAW264.7 macrophages were pretreated with dimethyl sulfoxide (DMSO) or 20 *μ*M PDTC for 1 h and then stimulated with LPS for 2 h. Steady state mRNA levels of TNF-*α* and IL-6 were examined by RT-PCR. (d) The levels of TNF-*α* and IL-6 mRNA were quantified by measuring band intensities and shown as fold increase relative to *β*-actin mRNA levels. Each value represents the means ± SD for *n* = 4. # and ∗ indicate statistically significant differences (*P* < 0.05) between propofol and LPS treated and LPS only treated groups, respectively.

A significant increase in TNF-*α* and IL-6 expression was observed after treatment with 100 ng/mL LPS for 1 h. However, 20 *μ*M pyrrolidine dithiocarbamate (PDTC) (Santa Cruz Biotechnology, Santa Cruz, CA), a selective chemical NF-*κ*B inhibitor, pretreatment for 1 h reduced the expression of TNF-*α* and IL-6 (Figures 4(a)–4(d)).

### 3.4. Effect of Propofol on LPS-Induced ROS Generation

**Figure 5:**
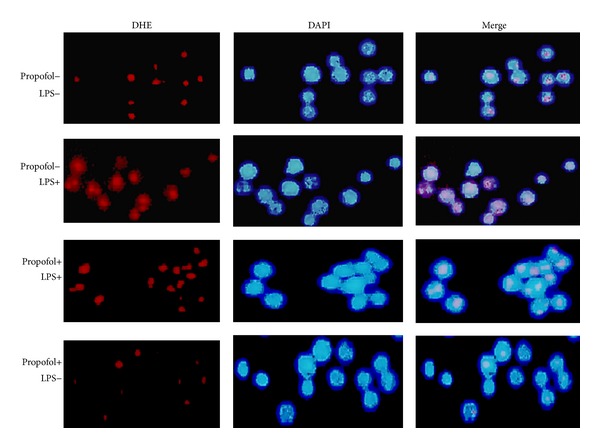
Effect of propofol pretreatment on LPS-induced generation of ROS. After a 40 min incubation following 50 *μ*M propofol or dimethyl sulfoxide (DMSO) treatment, cells were stimulated with LPS for 40 min then stained with 2 *μ*M dye hydroethidine (DHE) and incubated for 30 min at 37°C in the dark. Nuclei were stained with DAPI.

As expected, LPS (100 ng/mL) treatment significantly increased the intracellular ROS in macrophages. Remarkably, pretreatment with 50 *μ*M propofol significantly reduced LPS-induced increases in intracellular ROS. Again, propofol pretreatment alone did not alter levels of intracellular ROS (Figure 5).

### 3.5. Effect of Propofol on LPS-Stimulated NADPH Oxidase Activity

**Figure 6:**
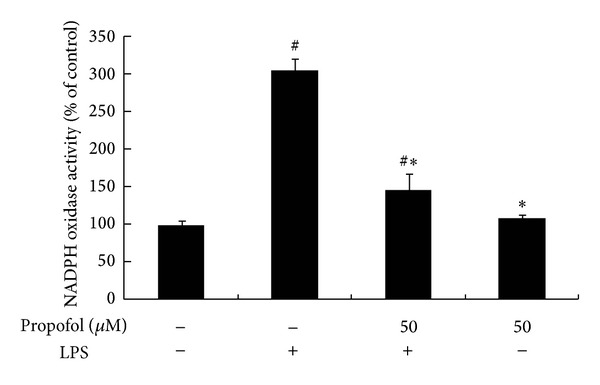
Effect of propofol pretreatment on NADPH oxidase activity. Cells were preincubated with dimethyl sulfoxide (DMSO) or propofol for 40 min, followed by 6 h incubation with or without LPS. The activity of NADPH oxidase was measured with an oxidase activity assay kit. Each value represents the means ± SD for *n* = 4. # and ∗ indicate that a value significantly (*P* < 0.05) differs from without both propofol and LPS or only LPS treated groups, respectively.

As shown in Figure 6, LPS treatment stimulated NADPH oxidase activity (298 ± 13.69%), while propofol pretreatment significantly lowered the degree of NOX stimulation (136 ± 13.69%). Treatment with 50 *μ*M propofol alone had no effect on NADPH oxidase activity.

### 3.6. Effect of Propofol on LPS-Induced NADPH Oxidase Expression

**Figure 7:**
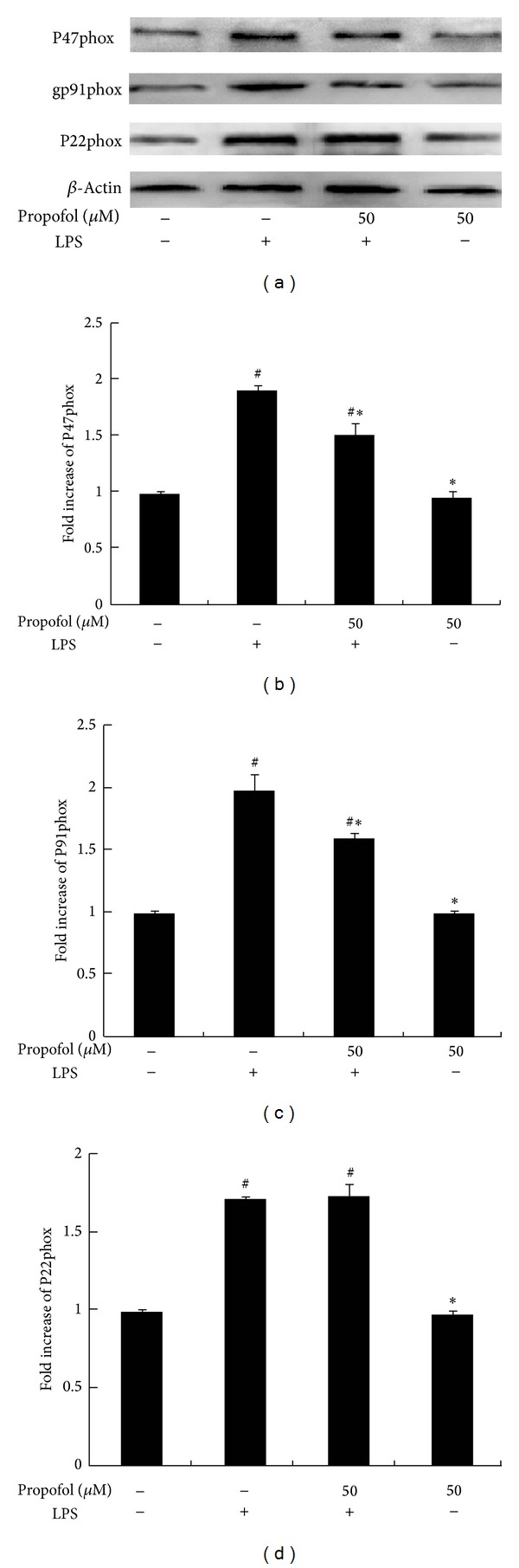
Effect of propofol pretreatment on LPS-induced NADPH oxidase expression. Cells were pretreated with dimethyl sulfoxide (DMSO) or propofol for 40 min and then stimulated with LPS for 8 h. (a) Protein expression of oxidase subunits was analyzed by Western blot analysis of whole cell lysates. (b, c, and d) The levels of subunit protein expression were quantified by measuring band intensities and displayed as fold increase relative to *β*-actin. Each value represents the means ± SD for *n* = 4. # and ∗ indicate that a value significantly (*P* < 0.05) differs from without both propofol and LPS or only LPS treated groups, respectively.

LPS (100 ng/mL) treatment for 8 h led to a significant increase in protein expression of NOX subunits (p47^phox^, gp91^phox^, and p22^phox^). Pretreatment with propofol effectively reduced the expression of p47^phox^ and gp91^phox^ in LPS-stimulated cells but had no effect on p22^phox^ expression. Propofol alone did not elicit effects on protein expression of oxidase subunits (Figures 7(a)–7(d)).

## 4. Discussion

Propofol is a general anesthetic that has anti-inflammatory and antioxidant effects [15]. Recent studies have focused on the inhibitory activities of propofol on LPS or inflammatory signaling—particularly the NF-*κ*B and ROS pathways [16, 17]. These studies have suggested that propofol directly inhibits ROS-mediated NF-*κ*B signaling. However, it remains unclear how propofol regulates ROS levels in macrophages. This report demonstrates that propofol treatment suppresses LPS-induced cellular inflammatory responses, NF-*κ*B signaling and ROS generation. Furthermore, propofol inhibits LPS-induced expression and activity of NADPH oxidase, which is the predominant source of ROS in macrophages.

Inflammation is a protective response by the body to ensure removal of detrimental stimuli, as well as a healing process for repairing damaged tissue [18, 19]. Macrophages play a critical role in initial recognition of microbial invasion and contribute to downstream immune responses by producing a variety of inflammatory mediators such as chemokines, cytokines (TNF-*α* and IL-6), and vasoactive amines [20, 21]. This response has been well characterized for microbial infections (particularly bacterial infections), which trigger receptors of the innate immune system including Toll-like receptors (TLRs). For example, TLR4 responds to LPS by releasing cytokines, such as TNF-*α* and IL-6 [22–24]. Our current study shows that propofol pretreatment significantly inhibits LPS-induced TNF-*α* and IL-6 expression in a dose-dependent manner. Our results suggest that propofol treatment leads to downregulation of the immune response.

Stimulation of TLR4 results in ROS production, which stimulates NF-*κ*B signaling. In turn, NF-*κ*B promotes the expression of cytokines (like IL-6 and TNF-*α*) and other immune mediators that participate in acute inflammatory responses [25]. ROS are critical for the innate immune response against intracellular bacteria and essential components of NF-*κ*B activation [26]. During an infection, high levels of ROS are targeted to sites of inflammation where they oxidize invading microbial pathogens and promote the expression of enzymes that damage surrounding tissue [27]. In addition to oxidation, ROS have immunomodulatory activity that can improve the immune response to an infection. In fact, ROS are considered second messengers in immune system signaling and act upstream of diverse biological responses involving transcriptional regulation, cytokines expression, and inflammation [28]. In this study, LPS-induced NF-*κ*B activation, as determined by Western blot analysis, and ROS production were significantly inhibited with pretreatment of clinically relevant concentrations of propofol. This suggests that propofol exhibits immunomodulatory effects through the reduction of NF-*κ*B phosphorylation and ROS levels.

NADPH oxidase activation plays an important role in LPS-induced innate immunity in macrophages by producing superoxide anions [26]. The subunits gp91^phox^ and p47^phox^ are essential components of NADPH oxidase. Mutations in these subunits are found in chronic granulomatous disease (CGD) and targeted deletions of these genes result in reduced bactericidal activity in mice [5, 29]. Chen et al. reported that treatment with 50 *μ*M propofol inhibited AngII-induced NADPH oxidase expression and activation in endothelial cells [30]. Propofol also abolished p47^phox^ upregulation in thoracic aortas [31]. The effect of propofol on NADPH oxidase expression in macrophages was not known. In this study, we showed that propofol remarkably inhibits LPS-induced NADPH oxidase activity and expression of p47^phox^ and gp91^phox^, but not p22^phox^, in macrophages [30, 31]. This finding is inconsistent with previous studies showing that propofol reduces gp91^phox^ and p22^phox^ expression in endothelial cells and p47^phox^ expression in aortic cells. We speculate that the different effects of propofol might be cell type dependent. Nevertheless, our study clearly demonstrates that propofol treatment inhibits NADPH oxidase activity and ROS production in macrophages. Notably, patients with chronic granulomatous disease fail to generate phagocyte-derived superoxide and related reactive oxygen intermediates, and our results suggest that propofol may have an effect in these patients by further increasing their risk of infection [18, 32].

In conclusion, the current study has demonstrated the immunomodulatory effects of propofol on macrophage cytokine and ROS production. Specifically, these include the inhibitory effects of propofol on LPS-induced TNF-*α* and IL-6 production and expression, as well as on NADPH oxidase expression and activity.
